# Computer-Aided Design of an Epitope-Based Vaccine against Epstein-Barr Virus

**DOI:** 10.1155/2017/9363750

**Published:** 2017-09-28

**Authors:** Julio Alonso-Padilla, Esther M. Lafuente, Pedro A. Reche

**Affiliations:** ^1^Barcelona Institute for Global Health (ISGlobal), Centre for Research in International Health (CRESIB), Hospital Clinic-University of Barcelona, Barcelona, Spain; ^2^Laboratory of Immunomedicine, Faculty of Medicine, University Complutense of Madrid, Ave Complutense S/N, 28040 Madrid, Spain

## Abstract

Epstein-Barr virus is a very common human virus that infects 90% of human adults. EBV replicates in epithelial and B cells and causes infectious mononucleosis. EBV infection is also linked to various cancers, including Burkitt's lymphoma and nasopharyngeal carcinomas, and autoimmune diseases such as multiple sclerosis. Currently, there are no effective drugs or vaccines to treat or prevent EBV infection. Herein, we applied a computer-aided strategy to design a prophylactic epitope vaccine ensemble from experimentally defined T and B cell epitopes. Such strategy relies on identifying conserved epitopes in conjunction with predictions of HLA presentation for T cell epitope selection and calculations of accessibility and flexibility for B cell epitope selection. The T cell component includes 14 CD8 T cell epitopes from early antigens and 4 CD4 T cell epitopes, targeted during the course of a natural infection and providing a population protection coverage of over 95% and 81.8%, respectively. The B cell component consists of 3 experimentally defined B cell epitopes from gp350 plus 4 predicted B cell epitopes from other EBV envelope glycoproteins, all mapping in flexible and solvent accessible regions. We discuss the rationale for the formulation and possible deployment of this epitope vaccine ensemble.

## 1. Introduction

Epstein-Barr virus (EBV), or human herpesvirus 4, is a large enveloped virus that belongs to the family herpesviruses *γ*. It has a size of 120–180 nm and a double-stranded linear DNA genome (~171 Kb long), encoding ~90 genes [1]. The genome is enclosed within a nucleocapsid protein which is in turn surrounded by a lipid envelope that contains the viral surface proteins essential for infection [2]. According to its expression, EBV genes are divided into immediate early (expressed very early during lytic infection, coding for transcription factors), early (interfere with the host metabolism and DNA synthesis), and late genes (including structural and nonstructural glycoproteins). There are two major subtypes of EBV (type 1 and type 2), which mainly differ in their nuclear antigen-3 gene (EBNA-3). Both types are detected all over the world, yet type 1 is dominant in most populations [3].

EBV is present in over 90% of the adult world population [4]. Most people become infected with EBV during childhood and develop little or no symptoms. However, if the infection occurs later in life, it can cause infectious mononucleosis (IM) in about 30–50% of the cases [5]. Viral transmission is primarily through saliva; hence, the nickname of kiss disease for IM. The virus can infect and replicate in epithelial and B cells. Infection of epithelial cells of the oropharynx has a relevant role in EBV expansion during primary infection [2]. However, B cells are the main targets of the virus. They are fundamental to establish an EBV infection—X-linked agammaglobulinemic patients are not infected by the virus [6]—and can pass the virus to epithelial cells by direct contact [7]. Moreover, it is in memory B cells that the virus persists as a long-term latent infection [8]. Tropism of EBV for B lymphocytes is mediated by cell surface molecules CD21 (i.e., complement receptor 2 (CR2)) and HLA-II that serve as receptors of the viral envelope glycoproteins gp350 and gp42, respectively [9]. Infection of B cells by EBV does not usually release viral progeny. Instead, the virus activates the cell cycle driving the expansion of latently infected B cells, inducing its own proliferation, thus getting persistently established in the lymphoid system [7, 8]. Latency is not permanent though, as EBV can periodically switch between latent and lytic states. Reactivation from latency is triggered by environmental stimuli and the process is tightly controlled by the immune system [10].

Immunity against EBV has been studied extensively [10, 11]. Natural killer (NK) cells play an important role in the innate immune response, delaying or preventing the EBV transformation of B cells through the production of interferon gamma (IFN-*γ*) [12]. Subsequently, the virus elicits strong adaptive immune responses, primarily mediated by cytotoxic CD8 T cells. CD8 T cell responses eliminate viral-infected cells upon recognition of EBV peptide antigens bound to MHC I molecules in the surface of target cells. Cytotoxic CD8 T cell response against EBV infection is so dramatic that, in IM patients, up to 50% of CD8 T cells recognize EBV-specific CD8 T cell epitopes, most derived from immediate early or early antigens [13, 14]. In contrast, CD4 T cell responses against the virus are less dramatic and focused [13]. CD4 T cells recognize peptide antigens bound to MHC II molecules and commit into different phenotypes of cytokine-producing T helper cells (Th) that control the immune response. Most EBV-specific CD4 T cells produce IFN-*γ* and tumor necrosis factor alpha (TNF-*α*), with a smaller number producing IL-2 which is the usual and expected Th1 antiviral response [15]. Regarding the humoral immune response, EBV infection triggers a potent reaction against various viral antigens. The acute primary infection is associated with the induction of IgM antibodies against the virus capsid antigen (VCA), which switches to an IgG isotype. IgG anti-VCA antibodies are not neutralizing and remain for life. Neutralizing IgG antibodies targeting viral major glycoprotein gp350 arise only after the resolution of the primary infection [16]. Other antibodies targeting non-neutralizing antigens (e.g., viral proteins located intracellularly) also appear sometime after the resolution of the primary infection [16, 17].

The immune system is capable of controlling EBV primary infection and reactivation phases, forcing the virus to stay latent in memory B cells. Such a control likely has a toll in the immune system. In fact, after extended periods of latency and being facilitated by its potent growth transforming capability, EBV appears to promote an increasing number of human cancers. Frequent cancers linked to EBV include several B cell malignancies, such as Burkitt's lymphoma (BL) and Hodgkin's lymphoma (HL), and epithelial cell malignancies, notably nasopharyngeal carcinoma (NPC) [18]. Furthermore, EBV infection has been implicated with autoimmunity and it is clearly a risk factor for developing multiple sclerosis and to a lesser systemic lupus erythematosus [19].

Currently, no medicine can cure EBV infection and there is no prophylactic or therapeutic vaccine against it. Clearly, a prophylactic vaccine against EBV will have a major impact in public health as it will prevent both EBV infection and related diseases [20]. In this study, we explored a reverse-vaccinology approach to design a prophylactic vaccine against EBV based on CD8 and CD4 T cell epitopes and B cell epitopes. For designing the T cell epitope vaccine component, we relied on combining legacy experimentation with bioinformatics analysis aimed to identify conserved and highly promiscuous T cell epitopes [21–23]. Given the size and complexity of EBV, we also introduced expression criteria to reduce the number of T cell epitopes and focus on those from early antigens with acknowledged function at the initial steps of primary infection [23, 24]. As for the B cell component, we included highly conserved experimentally determined B cell epitopes from EBV gp350 protein as well as potential B cell epitopes predicted in flexible solvent-exposed regions of other envelope proteins important for infection like gp42, gB, and gL. We are confident that our epitope vaccine ensemble poses a basis for developing a powerful and effective vaccine against EBV. Moreover, we trust that the approach and methods introduced in this work ought to become a paradigm of general use in reverse vaccinology.

## 2. Materials and Methods

### 2.1. Collection of EBV-Specific Epitopes

We retrieved experimentally defined EBV-specific T and B epitope sequences from the EPIMHC [25] and IEDB [26]. As inclusion criteria, we considered positive assays (excluding low-positive responses) and epitopes being linked to the course of a natural infection in humans for T cell epitopes and any human disease for B cell epitopes. We discarded duplicate peptides and when available, we also retrieved the MHC restriction elements of T cell epitopes. For B cell epitopes, we considered all unique sequences that were not included as part of longer peptides. In total, we obtained 247 unique B cell epitopes and 109 unique T cell epitopes (88 CD8 T cell epitopes and 21 CD4 T cell epitopes). These epitopes are available as supplementary data in Additional File S1 available online at https://doi.org/10.1155/2017/9363750, including Tables S1A, S1B, and S1C for CD8, CD4, and B cell epitopes, respectively. Perl scripts used to identify unique B and T cell epitopes from IEDB search outputs can be obtained from the corresponding author.

### 2.2. Generation of Clusters and Multiple Sequence Alignments of EBV Protein Sequences

We used CD-HIT [27] with default settings to generate clusters from 13,899 EBV protein sequences that included 89 translated coding DNA sequences (CDS) from a reference genome virus (accession: NC_007605). The protein sequences were downloaded following the links in the NCBI taxonomy database (TAX ID: 10376) [28]. We processed CD-HIT clusters with reference EBV proteins, removed identical sequences, and subsequently generated multiple sequence alignments (MSA) using MUSCLE [29]. As a result, we obtained 85 referenced MSA of EBV proteins that were used for further analysis. Software for clustering the sequences will be provided by the corresponding author upon written request.

### 2.3. Generation of EBV-Reference Proteome with Variable Sites Masked and Identification of Conserved Epitopes

We generated EBV-reference sequences with variable sites masked upon sequence variability analyses on the referenced MSA of EBV proteins. Briefly, we calculated the sequence variability in the MSA of EBV proteins using the Shannon entropy [30], *H*, as a variability metric [21, 24, 31]. Shannon entropy per site in a MSA is given by

(1) $$\begin{array}{c} H=-\overset{M}{\underset{i=1}{\sum }}P_{i}{\text{log}}_{2}P_{i}, \end{array}$$

where *Pi* is the fraction of residues of amino acid type *i* and *M* is equal to 20, the number of amino acid types. *H* ranges from 0 (total conservation, only one amino acid type is present at that position) to 4.322 (all 20 amino acids are equally represented in that position). We considered gaps as no data. To generate reference EBV consensus sequences, we assigned the computed variability, *H*, to the EBV-reference proteins included in the MSA and subsequently masked all positions with a variability, *H*, greater than 0.5 [32, 33]. We used this reference sequence to discard epitope sequences that did not match entirely with it. Hence, the epitopes that we considered conserved did not have a single residue with *H* > 0.5.

### 2.4. Prediction of Peptide HLA Presentation Profiles and Computation of Population Protection Coverage

T cells only recognize peptides when presented in the cell surface of antigen-presenting cells bound to HLA molecules (MHC molecules in humans). Therefore, we anticipated HLA presentation profiles of peptides by predicting peptide-HLA binding. For CD8 T cell epitopes, we predicted peptide binding using 55 HLA I-specific motif profiles [34–36]. A top 2% rank percentile was used to consider binding to the relevant HLA I molecule. For CD4 T cell epitopes, we predicted peptide binding to 15 reference HLA-DR molecules [37] using the IEDB binding tool [38]. We used a 5% percentile rank cutoff to consider that binding had occurred. The population protection coverage (PPC) of a set of epitopes is the proportion of the population that could elicit an immune response against any of them and can be computed by knowing the gene frequencies of the HLA I alleles that can present the epitopes [21]. For HLA I-restricted T cell epitopes, we used EPISOPT to compute epitope PPC [39]. EPISOPT uses HLA I allele frequencies for 5 distinct ethnic groups in the USA population (Caucasian, Hispanic, Black, Asian, and North American natives) [40] and can identify combinations of epitopes reaching a determined PPC in each of the population groups. We aimed to identify epitope combinations reaching a PPC of 95% in the 5 ethnic groups. For HLA II-restricted epitopes, we used IEDB PPC tool [41] to compute PPC for the world population using the epitope-HLA II presentation profiles predicted previously. We identified combinations of CD4 T cell epitopes reaching a maximum PPC by introducing into the IEDB PPC tool different combinations of epitopes with their corresponding HLA II binding profiles.

### 2.5. B Cell Epitope Prediction and Calculation of Flexibility and Solvent Accessibility

We considered flexible protein fragments identified in available 3D structures of the relevant antigens with relative solvent accessibility ≥ 50% as potential B cell epitopes. As residue flexibility values, we used normalized B factors, *Z*_*B*_ (2):

(2) $$\begin{array}{c} Z_{B}=\frac{B-\mu_{B}}{\partial_{B}}, \end{array}$$

where *B* is the residue B factor from the relevant PDB, *μ*_*B*_ is the mean of the *C_α_* residue of B factors, and ∂_*B*_ is the standard deviation of *C_α_* B factors. Flexible regions, potential B cell epitopes, consisted of 9 consecutive residues or more with flexibility equal or greater than the computed ∂_*B*_ (1.0). For each selected protein fragment, we obtained a flexibility score consisting of the average flexibility of the fragment residues and a solvent accessibility value consisting of the average relative solvent accessibility (RSA) of the residues. We obtained residue RSAs from the relevant PDB coordinates using NACCESS [42]. Solvent accessibility values and flexibility scores were computed in the same manner for experimental B cell epitopes.

### 2.6. Blast Searches, Protein Annotation, and Analysis Procedures

We mapped epitopes onto three-dimensional (3D) structures and retrieved UniProtKB [43] entries upon BLAST searches [44] against the PDB and Swissprot databases at NCBI (http://blast.ncbi.nlm.nih.gov/Blast.cgi). We also carried out BLAST searches with conserved epitope sequences as query against human proteins and human microbiome proteins to detect epitope identity to human or human microbiome proteins. These BLAST searches were carried out locally with standalone programs using an expectation value (−*e*) of 10,000. Human microbiome protein sequences for BLAST searches were obtained from the NIH Human Microbiome Project [45] at NCBI (https://www.ncbi.nlm.nih.gov/bioproject/43021). As human protein sequences, we used all human proteins available in the nonredundant (NR) collection at NCBI. We used PyMOL Molecular Graphics System, Version 1.8 Schrödinger, to visualize B cell epitopes on 3D structures. We identified function, subcellular localization, and temporal expression of selected EBV proteins (developmental stage) from UniProtKB [43].

## 3. Results

### 3.1. Reference EBV Proteome with Variable Residues Masked

Epitope-based vaccines can force the immune system to recognize conserved antigen regions. Therefore, a key step in our approach to epitope vaccine design is to carry out sequence variability analyses enabling the selection of conserved epitopes. To that end, we clustered all available EBV protein sequences around a reference EBV proteome (NC_007605), obtaining 85 protein clusters with EBV reference proteins on them (details in Materials and Methods). Upon aligning the sequences in the clusters, we subjected them to sequence variability analyses using the Shannon entropy, *H*, as variability metric. As a result, we identified that only 960 residue sites of the 42,998 evaluated had *H* ≥ 0.5 and generated reference consensus EBV sequences with those variable sites masked. A variability of *H* < 0.5 is a very stringent threshold for low variability and that only a few sites (960 residue sites) with *H* ≥ 0.5 were found indicates that EBV, as most dsDNA viruses, has a low mutation rate [1]. By matching EBV epitopes with this reference EBV proteome, we were able to select only those epitopes consisting of conserved residues (*H* < 0.5).

### 3.2. CD8 T Cell Epitope Component

To design the CD8 T cell vaccine component, we started with 88 unique EBV-specific CD8 T cell epitope sequences that were experimentally verified to be recognized in the course of a natural infection by EBV in humans. That set was reduced to 58 epitopes when we selected only those with a length of 9 residues (9 mers). We selected 9 mer peptides because most peptides presented by MHC I molecules are of that size [36]. Among those, we found 40 epitopes that did not have a single residue with *H* ≥ 0.5 and none were 100% identical to human proteins or human microbiome proteins (sequences and identity data included in Additional File S2, Table S2A). A strong CD8 T cell response to early antigens is key to clear the virus [14]. Therefore, after identifying the function and developmental stage of the relevant antigens in UniprotKB, we selected 16 CD8 T cell epitopes that were present in early antigens and had a reported functionality in primary EBV infection (Table 1). For each selected CD8 T cell epitope, we predicted its potential HLA I presentation profile (see Materials and Methods) and subsequently computed the population protection coverage (PPC) for 5 distinct ethnic groups present in the USA population (see Materials and Methods). PPC of CD8 T cell epitopes ranged from 5.08% to 57.84% (Table 1). Epitopes ARYAYYLQF and VSFIEFVGW had little PPC and were discarded for further analysis. Subsequently, we used EPISOPT [39] to identify epitope combinations within the remaining 14 CD8 T cell epitopes that could provide a PPC of 95% in each one of the ethnic groups. We found that just 5 epitopes were required to reach it. Moreover, we identified 40 different epitope combinations, 3 with 5 epitopes and 37 with 6 epitopes, that reached PPC ≥ 95% (data not shown). EPISOPT did not report more numerous epitope combinations because adding more epitope sequences did not increase the PPC [39]. The combination with only 5 epitopes that reached the largest PPC (96.0%) consisted of epitopes YVLDHLIVV, VLKDAIKDL, RVRAYTYSK, LPCVLWPVL, and AYSSWMYSY. However, the epitope combination that provided the highest PPC (97.1%) included 6 CD8 T cell epitopes: YVLDHLIVV, YRSGIIAVV, SVRDRLARL, RVRAYTYSK, LPCVLWPVL, and RRIYDLIEL. All the 14 CD8 T cell epitopes were found in at least one of the epitope combinations reaching 95% PPC. Subsequently, we considered all the 14 CD8 T cell epitopes for inclusion in the CD8 T cell vaccine component. The selected epitopes originate from 6 different viral antigens, including EBNA3, BRLF1, EBNA6, EBNA1, BMRF1, and BZLF1 (Table 1), and thus will also contribute to a multiantigenic response.

### 3.3. CD4 T Cell Epitope Component

We identified a total of 21 EBV-specific CD4 T cell epitopes from the relevant epitope databases that were elicited in the course of a natural infection by EBV in humans (Table S1B in Additional File S1). Of those, we selected 10 epitopes that were conserved (Table 2) and none were 100% identical to human proteins or human microbiome proteins (see Table S2B in Additional File 2). The size of the conserved CD4 T cell peptides ranged from 15 to 20 residues long. We next identified their HLA II presentation profile by predicting peptide-MHC II binding to 15 distinct HLA-DR molecules that are frequently expressed in the population (see Materials and Methods). We chose to target HLA-DR molecules for two reasons: the alpha chain is nonpolymorphic [32] and HLA-DR are expressed at a much higher density in the cell surface of antigen-presenting cells than any other HLA II molecules [46] and thus are more relevant for epitope vaccine design [47].

Upon determining epitope HLA II presentation profiles, we computed the PPC for the world population as indicated in Materials and Methods. The maximum PPC that could be reached by considering the entire set of HLA-DR molecules is 81.81%. The PPC of selected CD4 T cell epitopes ranged from 0% (QKRAAPPTVSPSDTG) to 69.85% (MLGQDDFIKFKSPLV). The PPC that could be reached by combining all distinct HLA-DR molecules that were found to bind the selected CD4 T cell epitopes was 81.81% (Table 2). This PPC was reached by considering only the epitopes MLGQDDFIKFKSPLV, AGLTLSLLVICSYLFISRG, SRDELLHTRAASLLY, and PPVVRMFMRERQLPQ derived from antigens BFRF1, BHRF1, BARF1 and EBNA6, respectively. Antigens BFRF1 and EBNA6 are nuclear proteins, whereas BARF1 is a secreted protein and BHRF1 is a membrane-bound antigen. We considered this 4-epitope combination as the optimal CD4 T cell vaccine component.

### 3.4. B Cell Epitope Component

We assembled the B cell epitope vaccine component from a set of 247 EBV-specific unique linear B cell epitope sequences ranging from 4 to 38 amino acids (Table S1C in Additional File S1). From those, we discarded B cell epitopes shorter than 9 residues and kept 117 that were conserved with no single residue with *H* > 0.5 (details in Materials and Methods). Moreover, none of these 117 B cell epitopes were identical to human proteins or to human microbiome proteins (data provided in Additional File S2, Table S2C). We analyzed the subcellular location of selected antigens to identify those that are expressed in the viral surface, accessible for antibody recognition. We found that the vast majority of the selected epitopes originated from viral intracellular antigens and therefore have no interest for B cell epitope vaccine design. We only found 9 B cell epitopes that were present in viral envelope glycoproteins: 7 from the major surface antigen gp350, the main viral determinant mediating viral attachment to B cells [48] and 2 from the envelope glycoprotein B (gB), key for the fusion of viral and host cell membranes during viral entry [49] (Table 3). However, only the 7 gp350 B cell epitopes mapped on the protein ectodomain and were further considered for the B cell epitope vaccine component. The 2 gB epitopes, QKRAAQRAAGPSVAS and VSGFISFFKNPFGGM, mapped onto the inner and transmembrane regions, respectively (Table 3).

Flexible and accessible linear B cell epitopes are often cross-reactive with antibodies against native antigens and are thereby of prime interest for epitope vaccine design [50]. Therefore, to further analyze the suitability for vaccine design of the 7 remaining gp350 B cell epitopes, we devised a system to quantify the flexibility and solvent accessibility of B cell epitopes from the known 3D structures. Briefly, we used normalized B factors and relative residue solvent accessibility computed from the relevant PDBs as measures of flexibility and accessibility (details in Materials and Methods). Following these criteria, we discarded the gp350 B cell epitope PSTSSKLRPRWTFTSPPVTT, for it mapped onto a region of the gp350 without a 3D structure and we could not readily evaluate its flexibility and accessibility. Of the 6 gp350 B cell epitopes that mapped onto the available gp350 3D structure (PDB: 2H6O), only 3 of them, SKAPESTTTSPTLNTTGFA, YVFYSGNGPKASGGDYCIQS, and QNPVYLIPETVPYIKWDN, had flexibility and solvent accessibility values supporting that they were readily accessible for antibody recognition (Table 3). In fact, visual inspection of epitopes SVKTEMLGNEID and QVSLESVDVYFQDVFGTMWC in the gp350 3D structure revealed that they were buried and thus not accessible for antibody recognition, while B cell epitope TNTTDITYVGD though accessible (60%) was located in a rigid region of the protein (Figure S1 in Additional File S3). These epitopes will likely induce antibodies that will be unable to recognize native antigens and were discarded from the B cell vaccine component.

**Figure 1:**
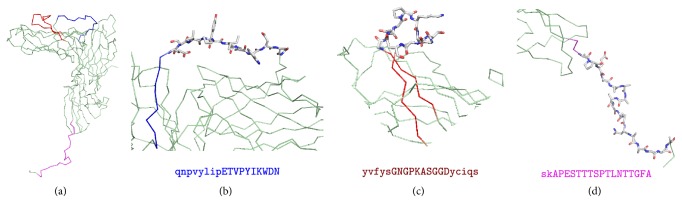
Structural mapping of selected experimental EBV-specific B cell epitopes. Conserved EBV epitopes map onto two different regions of the 3D structure of gp350 (PDB code: 2H6O): QVNYLIPETVPYIKWDN and YVFYSGNGPKASGGDYCIQS map at the glycan-free surface of the CR2 receptor binding site; SKAPESTTTSPTLNTTGFA maps at the C-term tail of the PDB. (a) General view of gp350 featured as ribbon with B cell epitopes highlighted in red, blue, and purple. Protein regions of the selected epitopes are zoomed in panels (b, c, d). We show in sticks the part of the epitopes that exhibited greater flexibility and accessibility which was ultimately selected for the proposed vaccine ensemble. In ribbon, we show the B cell epitope residues that do not comply with the flexibility and accessibility criteria (typed in a minor case in the corresponding sequence indicated at the bottom of each panel). Figures were rendered using PyMOL.

Following the hypothesis that highly flexible protein regions are suitable B cell epitopes for epitope vaccine design, we identified inner antigenic regions in the gp350 B cell epitopes SKAPESTTTSPTLNTTGFA, YVFYSGNGPKASGGDYCIQS, and QNPVYLIPETVPYIKWDN (APESTTTSPTLNTTGFA, GNGPKASGGD, and ETVPYIKWDN, resp.), encompassing only residues with a high degree of flexibility (≥1.0) and solvent accessibility greater than 50% (Table 3). Visual inspection of the gp350 B cell epitopes in the 3D structure clearly showed that the selected core fragments were located in highly flexibly and accessible regions of the structure while some parts of the remaining epitope were buried or semiburied (Figure 1()). Therefore, we regarded the antigenic core regions (APESTTTSPTLNTTGFA, GNGPKASGGD, and ETVPYIKWDN) identified in the gp350 B cell epitopes as the experimental B cell component of the EBV epitope vaccine ensemble.

**Figure 2:**
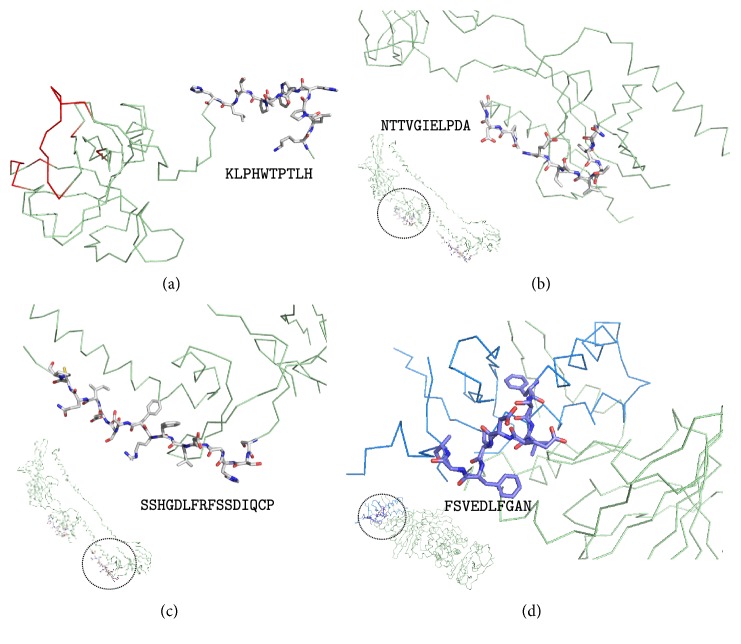
Structural mapping of predicted B cell epitopes in EBV envelope proteins. (a) KLPHWTPTLH in EBV gp42 3D structure (PDB: 3FD4 chain A); epitope shown as sticks and gp42 region interaction with HLA-DR is shown in red. (b) NTTVGIELPDA and (c) SSHGDLFRFSSDIQCP at EBV gB 3D structure (PDB: 3FVC) map, respectively, in its domain II and domain III; epitopes shown as sticks. (d) FSVEDLFGAN at gL 3D structure (PDB: 5T1D chain B) in its domain I (colored in blue); gH is colored in pale green. In (b, c, d), the corresponding whole structure is shown minimized at the bottom left of each panel; the magnified epitope mapping region is circled in them. In (a, b, c), the protein backbone is featured as pale green ribbon. Figures were rendered using PyMOL.

As all experimental B cell epitopes suitable for epitope vaccine design were in gp350, we sought to identify potential B cell epitopes from the 3D structures of EBV envelope proteins gp42 (PDB: 3FD4), gB (PDB: 3FVC) and the heterodimer conformed by gH and gL (PDB: 5T1D). These proteins have been described to participate in the viral attachment and/or fusion to the host cell membrane required for viral entry [49, 51, 52]. We considered as potential B cell epitopes, antigen fragments in the relevant 3D structures consisting of 9 or more consecutive residues with flexibility ≥ 1.0 and an average accessibility ≥ 50% (details in Materials and Methods). As a result, we identified a potential B cell epitope in gp42 protein (KLPHWTPTLH), two at the gB protein (NTTVGIELPDA and SSHGDLFRFSSDIQCP), and one in the gL monomer (FSVEDLFGAN) (Table 4). No epitopes fulfilling the required criteria were identified at the gH protein. These predicted B cell epitopes were mapped to their corresponding 3D structures to confirm that they were in readily accessible regions for antibody recognition (Figure 2()). KLPHWTPTLH mapped at the N-terminal region of gp42, which is involved in gH interaction and sits opposite to the HLA-DR binding site of the molecule (colored in red in Figure 2(a)). The gB epitopes mapped onto two distinct regions, domains II and III, that are likely relevant for interaction with other glycoproteins involved in viral entry [49] (Figures 2(a) and 2(b)). The single gL epitope mapped in a region in close proximity to gH and the binding site of a monoclonal antibody (mAb) EID1 that interferes with EBV infection of epithelial cells [52] (Figure 2(d)). We also verified that none of the predicted B cell epitopes were identical to human proteins or human microbiome proteins (Table 4).

## 4. Discussion

Over 90% of human adults are infected with EBV. Most infections occur in childhood and are asymptomatic or course with nonspecific symptoms. Nonetheless, EBV is the primary cause of IM when infection occurs in early adulthood. Furthermore, the viral infection is associated with autoimmunity and a number of lymphocyte and epithelial cell malignancies [18, 19]. Despite its wide impact, there is no treatment available, hence the growing interest in finding a prophylactic and/or therapeutic EBV vaccine.

The target population for an EBV prophylactic vaccine in the developed world would be 10- or 11-year-old children, before they are susceptible to most severe IM symptomatologies. It is acknowledged that by precluding the initial viral infection, the risks of developing EBV-associated autoimmune and cancer disorders would also be reduced [53]. In sub-Saharan Africa and southern China, where Burkitt's lymphoma and nasopharyngeal carcinoma are major public health problems and children are infected by EBV earlier in life, the vaccine target would be much younger infants. EBV-naïve transplant recipients susceptible to suffer posttransplant lymphoproliferative disorders (PTLD) would also benefit from a prophylactic vaccine [54].

Currently, the most advanced EBV vaccine clinically tested consists of a gp350 subunit that was administered with AS04 adjuvant to virus-naïve young adults [55]. The gp350 subunit vaccination strategy follows the approach successfully used in other viral infections, that is, induction of neutralizing antibodies (nAbs) against the most abundant glycoprotein on the virus, which also represents the main target of naturally occurring nAbs [16]. In this regards, a microneutralization assay based on an EBV expressing green-fluorescent protein has been very recently developed to provide measurement of humoral EBV vaccine responses in large clinical trials [56]. Another EBV vaccine trial was designed to control the expansion of EBV-infected B cells, based on the generation of CD8 T cell immunity to EBNAs [57]. Specifically, the vaccine consisted of a single EBNA3A epitope restricted by HLA-B08 administered as a peptide along with tetanus toxoid as adjuvant [57].

A major outcome of the Sokal et al. [55] clinical trial was that immunization with gp350 did not protect from new viral infections [55]. Therefore, it has been suggested that a prophylactic vaccine against EBV should elicit B cell responses also against all 5 major viral envelope proteins involved in host-cell attachment and entry, including gp42, gH, gL, BMRF2 (gp350), and gB [58]. Among these, at least the first four are known to elicit neutralizing antibodies [59]. The induction of cytotoxic T cell responses against early viral antigens has been as well suggested in order to destroy recently infected B cells [14, 53, 59]. Attaching to these premises, we used a computer-assisted strategy to design a prophylactic epitope vaccine ensemble against EBV infection.

**Figure 3:**
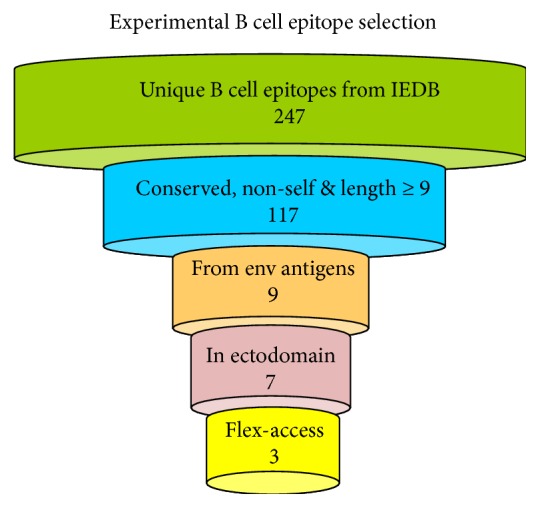
Strategy for experimental B cell epitope selection. Overview of the approach devised to select invariant experimental EBV-specific B cell epitopes for the B cell component of an epitope-based vaccine against EBV. The approach comprises 5 steps: (1) selection of unique epitopes from databases; (2) sequence variability filtering and testing for self-peptides; (3) selection of epitopes from viral envelope antigens; (4) progression of epitopes located to envelope protein ectodomains; (5) final output of epitopes that fulfill the flexibility and accessibility criteria established in the text. None of the epitopes that we selected were identical to human proteins or proteins from the human microbiome.

The strategy that we followed to design the EBV vaccine relied on combining legacy experimentation consisting of experimentally defined epitopes with immunoinformatics predictions. This strategy was first conceived to assemble CD8 T cell epitope vaccines [21, 39] and latter extended to include CD4 T cell epitope vaccines [22]. The main advantage of this approach is that of saving time and resources as it mainly relies on experimentally validated epitopes, not on predicted epitopes, using immunoinformatics to identify those that are more suitable for epitope vaccine design. It is worth noting that epitope prediction is not a precise science and epitope prediction methods only facilitate epitope discovery by providing candidates that need to be validated experimentally. Therefore, our strategy ought to gain widening acceptance as a vaccine design tool whenever ample experimental epitope data is readily available. Key criteria for epitope inclusion/selection are conservation and binding to multiple MHC molecules for maximum population protection coverage. Here, we also added that the source of CD8 T cell epitopes had to be from early EBV antigens with defined function in the primary infection process. Moreover, we checked that peptides were nonself and did not have exact matches with human proteins or human microbiome proteins and extended the approach to B cell epitopes. To that end, we devised a system to select from experimentally defined B cell epitopes those that were conserved, nonself and located on the ectodomains of viral envelope antigens and consisted of highly flexible and solvent-accessible residues (Figure 3()). Note that we are not discriminating B cell epitopes from non-B cell epitopes in primary sequences. In fact, solvent accessibility or flexibility alone cannot discriminate B cell epitopes from non-B cell epitopes in primary sequences [60]. Instead, we are selecting known B cell mapping in the antigen surface that isolated from the antigen context can elicit antibodies cross-reacting with the native antigens and hence are worth for epitope vaccine design [61].

The composition of the epitope vaccine ensemble designed in this study includes 14 CD8 T cell epitopes, 4 CD4 T cell epitopes, and 7 B cell epitopes (Table 5). None of these epitopes matched exactly to human proteins or human microbiome proteins. This result is somewhat predictable for we focused mostly on epitopes that have been verified experimentally and it should be expected that the immune system selected nonself targets for recognition. Nonetheless, a few of the selected epitopes have a high identity with human microbiome proteins (around 88.9%, Table 5). Whether this high identity to human microbiome proteins could be a source of trouble is arguable: detection of epitope identity to self-proteins required using BLAST with expectation values of 10000, epitope matches may not be available for recognition, and epitope recognition can be disrupted by single amino acid changes.

According to some authors, the ideal EBV CD8 T cell epitope component should include antigens EBNA2, EBNA-LP, and BHRF1, which are abundant at the very initial stage of B cell infection [14]. Our epitope vaccine ensemble does not include CD8 T cell epitopes from these three antigens. However, it includes CD8 T cell epitopes from other EBV early antigens, such as EBNA1, EBNA3, EBNA6, BMRF1, BRLF1, and BZLF1 (Tables 1 and 5). Although a 95% PPC was reached with just 5 CD8 T cell epitopes, the key importance of a broad multiantigenic cytotoxic response prompted us to incorporate 14 CD8 T cell epitopes. For the CD4 T cell component, our proposed vaccine ensemble includes 4 epitopes reaching the maximum PPC possible of 81.8% provided by the reference set of HLA II molecules targeted for binding predictions [37]. The PPC of the CD4 T cell component is likely an underestimation. HLA II molecules are very promiscuous [62] and the selected epitopes will surely bind and be presented by other HLA II molecules not included in the selected reference set [37].

For the B cell epitope vaccine component, we included 7 B cell epitopes consisting of 3 experimental B cell epitopes from gp350 plus 4 other predicted B cell epitopes from EBV envelope proteins gp42, gB, and gL, all of them continuous and with high flexibility and solvent accessibility. We focused on linear B cell epitopes because they can be delivered isolated from their antigen context to induce selective humoral responses. We sought to predict B cell epitopes on gp42, gB, and gL that can be used to elicit antibodies that are cross-reactive with the native antigens. To that end, we needed to identify solvent-exposed B cell epitopes in the mentioned antigens and we could have used a number of methods to predict conformational B cell epitopes from the available 3D structures (reviewed in [63]). However, conformational B cell epitopes can not be isolated from their protein context and used as immunogens. Therefore, we turned our attention to linear B cell epitopes as they can be delivered isolated from the antigen and induce selective humoral responses. There are also a number of methods to predict linear B cell epitopes from primary sequences (reviewed in [60, 64]), but the predicted epitopes seldom match in solvent-accessible regions and are notoriously unreliable [60, 65, 66]. Hence, in this study, we assumed that highly flexible and solvent-accessible fragments in protein surfaces are potential linear B cell epitopes [50] and devised a system to identify them from the relevant 3D structures (details in Materials and Methods). Specifically, predicted B cell epitopes consisted of conserved fragments with at least 9 consecutive residues with flexibility (normalized B factor) > 1 and an average relative solvent-exposed accessibility ≥ 50%.

Analysis of the structural mapping of the selected B cell epitopes onto the relevant 3D structure can reveal their importance for epitope vaccine design. The gp42 B cell epitope (KLPHWTPTLH) is located in the N-terminal portion of the protein far and opposite from the HLA-DR contact region (Figure 2(a)). Therefore, antibodies against this gp42 B cell epitope will unlikely block the gp42 interaction with HLA-DR required for viral entry into B cells. The gp42 N-terminal region, where KLPHWTPTLH maps, interact with gH at a site in close proximity to the *β*1-integrin-binding motif “KGD” [52]. Both gp42 and peptides from the N-terminal region of gp42 that binds to gH interfere with *β*1-integrin interaction and viral entry in epithelial cells [52]. In this context, the role of antibodies against this gp42 epitope with regard to viral entry in epithelial cells is unclear. Binding of antibodies to the epitope when gp42 is in complex with gH could prevent epithelial infection by EBV. However, such prevention is unlikely if antibodies against the epitope block the interaction between gp42 and gH. Despite poor neutralizing qualities of the gp42 B cell epitope KLPHWTPTLH, antibodies against it could still contribute to viral clearance by promoting complement activation and phagocytosis. The two predicted B cell epitopes in gB, NTTVGIELPDA, and SSHGDLFRFSSDIQCP, mapped onto two distinct protein domains (Figures 2(b) and 2(c)) that are thought to be relevant in the mechanism of EBV fusion to host membranes [49]. Hence, antibodies binding at this region could interfere in the vital fusion step required for viral entry. The B cell epitope predicted in gL, FSVEDLFGAN, mapped onto a region intertwined with gH and is in close proximity to the binding site of mAb E1D1 [52]. This antibody has been described to inhibit gH fusion to epithelial cells despite locating far from the gH integrin binding site (KGD). Whether an antibody against gL-protruding epitope FSVEDLFGAN might also exert a similar distant effect is unknown but remains a possibility.

**Figure 4:**
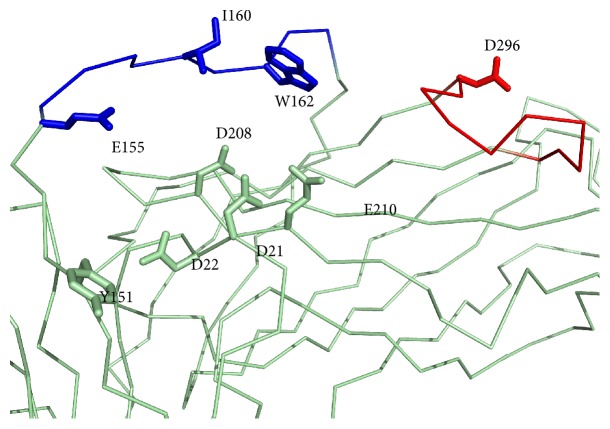
The EBV gp350 contact region with CR2. EBV B cell epitopes ETVPYIKWDN and GNGPKASGGD map onto a gp350 region that interacts with CR2; epitopes colored blue and red and the gp350 backbone featured as pale green ribbon. Side chains of the residues described to interact with CR2 receptor by Young et al. [67] are shown as sticks. Figure was rendered using PyMOL.

Flexibility and accessibility were also key criteria to select and refine experimental B cell epitopes, leading to the selection of the gp350 B cell epitopes ETVPYIKWDN, GNGPKASGGD, and APESTTTSPTLNTTGFA (Table 3 and Figure 1()). Two of these B cell epitopes, ETVPYIKWDN and GNGPKASGGD, mapped onto the glycan-free region of gp350 described to interact with the CR2 receptor [48]. Furthermore, residues E155, I160, and W162 from ETVPYIKWDN and D296 from GNGPKASGGD have been shown to contact the CR2 receptor (Figure 4()) [67]. Noteworthy, the well-characterized EBV nAb 72A1 binds to gp350 in this glycan-free region [67]. Therefore, B cell epitopes ETVPYIKWDN and GNGPKASGGD have a great potential to induce neutralizing antibodies. In fact, GNGPKASGGD and ETVPYIKWDN are within peptide fragments that have been shown already to elicit antibodies that block binding of mAb 72A1 to gp350 [68]. Lastly, epitope APESTTTSPTLNTTGFA mapped onto the C-terminal end of the solved structure of gp350 (Figure 1()). Mutagenesis of its E425 and S426 residues did not inhibit binding of gp350 to mAb 72A1 [48]. Although initially far from the receptor interaction region and containing a glycosylated asparagine residue (N435), it cannot be discarded that an antibody targeting it could help to control viral infection, for example through antibody-mediated complement activation and phagocytosis. Overall, these results validate the conservancy, flexibility, and accessibility criteria followed for the selection and prediction of B cell epitopes.

We trust that the application of the knowledge-based approach depicted in this work to design an epitope vaccine ensemble against EBV can save time and effort developing such a vaccine, as most of the components consist on experimentally defined EBV-specific epitopes. However, our epitope-based vaccine ensemble is theoretical, and extra validations will be required prior to formulating a vaccine that can actually be tested. For example, T cell epitopes used in our vaccine have been shown to be immunogenic in the context of experimentally defined HLA restriction elements (see Tables 1 and 2). However, we predicted that these epitopes will be also immunogenic in the context of different HLAs. To test that, T cells from subjects expressing the relevant HLA molecules can be expanded using dendritic cells loaded with the corresponding epitope peptides and cloned. Subsequently, T cell clone immunoreactivity can be checked through a number of assays (ELISPOT, intracellular cytokine staining, etc) using B-LCL 721.221 cells expressing single HLA molecules as described elsewhere [21, 69]. Selected B cell epitopes should also be subjected to extra validations, in particular to test whether they elicit antibodies cross-reacting with native antigens. To that end, sera from immunized mice with B cell epitope peptides could be used to check whether they recognize native antigens in ELISA assays and/or interfere with EBV infection of epithelial and B cells as described elsewhere [68]. Once the individual components of the epitope vaccine ensemble had passed experimental validation, it will still remain to elucidate how to formulate such a vaccine for delivering the epitopes.

There are several choices to formulate epitope vaccines ranging from peptide-based formulations to genetic formulations. Regardless of the choice, CD4 T cell epitopes need to be physically linked with the other selected epitopes, particularly B cell epitopes, to elicit productive Th cells [70]. A peptide-based vaccine has already been tested for the delivery of an EBV CD8 T cell epitope fused with tetanus toxoid to increase immunogenicity and elicit Th responses [57]. Similarly, a polymeric epitope concatemer in the form of a “string-of-beads” could be chemically synthesized or formulated as a genetic construct [71]. In either cases, the order of the epitopes and the presence of cleavage sites between them are crucial features to address [71]. Concatenating epitopes can result in toxic products and tools to predict toxicity can also be used to optimize epitope concatemers [72]. Toxicity of epitope vaccine formulations should nevertheless be checked in cellular assays prior to carrying out any immunization studies. In general, poor immunogenicity is an important issue with peptide-based formulations [22]. A recent development in vaccine formulation that increases the immunogenicity of the epitope-peptide components consists in the use of nanoparticles of diverse nature [73]. For example, Kuai et al. [74] used high-density lipoprotein-mimicking nanodiscs coupled with peptides to stimulate potent tumor-specific CD8 T cell responses that inhibited tumor growth in a murine model of colon carcinoma. Nanoparticles have also been used to deliver genetic constructs, particularly RNA constructs. RNA-based vaccine formulations offer lower safety concerns and enhanced immunogenicity with regard to those based on DNA, and inherent RNA instability can be overcame using nanoparticles for delivery [75].

Ideally, the B cell response should only be focused on B cell epitopes. To that end, a solution would be formulating the epitope vaccine as liposomal or virosome-like particles, where the selected T cell epitopes, either alone or concatenated, ought to be placed encapsulated inside the particle and the B cell epitopes displayed linked in the outer part of the particle [76, 77]. These liposomal vaccine formulations are also more immunogenic than those consisting of genetic or synthetic peptide-based constructs [76, 77]. Moreover, immunogenicity can be further enhanced by the inclusion of appropriated adjuvants [78].

Epitope vaccine formulations, as any vaccine candidate, should be evaluated in preclinical animal models prior to clinical testing in humans. However, in the case of EBV, this stands as a major drawback as there is a lack of appropriate animal models that recapitulate EBV infection and its immune control [79]. Thus, EBV vaccine immunogenicity and protection capabilities have to be assessed in clinical studies. Although this is very informative and may accelerate the developmental process, it also carries high associated costs early in the discovery path and involves enrollment of participants, which is not at the reach of many research groups. The clinical status of the target population to test EBV prophylactic vaccine candidates should also be considered. For instance, the phase II study by Sokal et al. [55], the most advanced of any EBV vaccine tested so far [54], involved a total of 181 EBV-seronegative, healthy, young volunteers between 16 and 25 years of age that were randomized in a double-blind fashion to receive either placebo or a recombinant EBV subunit glycoprotein 350.

## 5. Conclusions and Limitations

EBV infection is associated with a number of human diseases, including cancer and autoimmunity. Currently, it is unclear why some individuals with apparently proper responses to EBV develop associated diseases while others do not, but surely genetic and environmental factors, including life style and past pathogen encounters, play a role [80–82]. In any case, a prophylactic EBV vaccine will be beneficial in preventing EBV-associated diseases [53, 59]. We herein provide an epitope ensemble that would serve to develop an epitope-based prophylactic vaccine against EBV infection, eliciting both adaptive cellular and humoral immunity. The T cell component consists of highly conserved experimental EBV-specific epitopes capable of eliciting cellular responses in virtually the whole population. The B cell component consists of conserved experimental and predicted B cell epitopes from EBV envelope proteins gp350, gp42, gB, and gL. These epitopes were selected from the relevant 3D structures applying a novel structure-based reverse vaccinology approach that includes calculation of flexibility and solvent accessibility values. As a result, we identified B cell epitopes that could elicit antibodies interfering with EBV entry in epithelial and B cells. Whether our epitope vaccine ensemble has also any therapeutic value is arguable but, clearly, it is harder to combat EBV once it has established a latent infection.

This study has limitations that may handicap its translation into an EBV vaccine. Appropriate antigen processing is a key limiting factor in the immunogenicity of T cell epitopes [83]. Therefore, we selected experimental T cell epitopes that were shown to be processed and presented in the course of a natural infection with EBV and assumed that T cell epitope immunogenicity will be then only determined by their binding to MHC molecules. This assumption has not been thoroughly tested and it is very sensitive to possible errors in the databases where we collected the data. In the same line, population coverage estimates for the T cell component need to be tested as they are inferred from peptide binding predictions to MHC molecules. Nonetheless, the reliability of peptide-MHC binding predictions has been widely proved [84]. With regard to the B cell component, we deliberately failed to include conformational epitopes as they cannot be isolated from their context and solely focused on linear B cell epitopes. Whether these B cell epitopes are able to elicit antibodies recognizing the native protein conformations needs to be tested.

## Supplementary Material

Additional File S1. Experimentally determined EBV-specific T and B cell epitopes. EBV-specific epitopes are included in three tables: Table S1A (CD8 T cell epitopes), Table S1B (CD4 T cell epitopes) and Table S1C (B cell epitopes). Tables are depicted in three sheets of the excel file. Additional File S2. Conserved EBV-specific CD8 T, CD4 T and B cell epitopes. Conserved epitopes are included in three tables: Table S2A (Conserved EBV-specific CD8 T epitopes), Table S2B (Conserved EBV-specific CD4 T epitopes) and Table S2C (conserved EBV-specific B cell epitopes). Tables are depicted in three sheets of the excel file. Additional File S3. Figure S1. Structural mapping of experimental gb350 B cell epitopes that were discarded. Figure depicts the location in the relevant 3D-structure of conserved gb350 B cell epitopes that were discarded for epitope-vaccine design. Conserved EBV B cell epitopes SVKTEMLGNEID and QVSLESVDVYFQDVFGTMWC were discarded because mapped onto buried or semi-buried regions of gp350 (PDB code: 2H6O). The conserved EBV epitope TNTTDITYVGD was discarded because mapped onto a highly structured and rigid region. The gp350 3D-structure is shown as a pale green ribbon backbone but the epitopes that are shown as sticks. Figure was rendered using PyMOL.

## Figures and Tables

**Table 1:** Conserved EBV-specific CD8 T cell epitopes from early antigens.

Epitope	Antigen gene	AN^1^	HLA I restriction^2^	Predicted HLA I profile	PPC%^3^
RPPIFIRRL	EBNA3	P12977	B^∗^07, B^∗^08, B^∗^0702	B^∗^0702, B^∗^0801, B^∗^3501, B^∗^5101, B^∗^5102, B^∗^5103, B^∗^5301, B^∗^5401, C^∗^0102	57.84
SVRDRLARL	EBNA3	P12977	A^∗^0201	A^∗^0201, A^∗^0203, A^∗^0206, A^∗^0214, B^∗^0702, B^∗^0801, B^∗^1517	56.66
YVLDHLIVV	BRLF1	P03209	A^∗^0201, A^∗^02	A^∗^0201, A^∗^0202, A^∗^0203, A^∗^0204, A^∗^0205, A^∗^0206, A^∗^0209, A^∗^0214, B^∗^1517, B^∗^5701	47.34
QPRAPIRPI	EBNA6	P03204	B^∗^0702	B^∗^0702, B^∗^3501, B^∗^5101, B^∗^5102, B^∗^5103, B^∗^5301, B^∗^5401, B^∗^5502	43.56
LPCVLWPVL	BZLF1	P03206	B^∗^0702	B^∗^0702, B^∗^3501, B^∗^5101, B^∗^5102, B^∗^5103, B^∗^5301, B5401	42.4
RVRAYTYSK	BRLF1	P03209	A^∗^0301, A^∗^03	A^∗^0301, A^∗^1101, A^∗^3101, A^∗^3301, A^∗^6801	41.46
AYSSWMYSY	EBNA3	P12977	A^∗^30	A^∗^0101, B^∗^2701, C^∗^0702	36.38
VLKDAIKDL	EBNA1	P03211	A^∗^0203	A^∗^0203, A^∗^0204, A^∗^0205, A^∗^0206, A^∗^0207, A^∗^0214, B^∗^0801, C^∗^0304	33.72
QAKWRLQTL	EBNA3	P12977	B^∗^08	B^∗^0702, B^∗^0801, B^∗^1400, C^∗^0102	32.48
RRIYDLIEL	EBNA6	P03204	B^∗^2705	B^∗^1400, B^∗^2702, B^∗^2703, B^∗^2704, B^∗^2705, B^∗^2706, B^∗^2709, C^∗^0702	30.42
RLRAEAQVK	EBNA3	P12977	A^∗^03, A^∗^0301	A^∗^0301, A^∗^1101, B^∗^1513	28.7
CYDHAQTHL	BMRF1	P03191	A^∗^2402	A^∗^0207, A^∗^2402, B^∗^3801	27.3
SENDRLRLL	BZLF1	P03206	B^∗^4002, B60	B^∗^4002, B4402	14.18
YRSGIIAVV	BMRF1	P03191	B^∗^3906, Cw6	A^∗^0202, A^∗^0203, A^∗^0204, A^∗^0205, A^∗^0209, B^∗^1509, B^∗^1510, B^∗^1516, B^∗^2709, B^∗^3801, B^∗^39,011, B^∗^3909	12.82
ARYAYYLQF	DBP	P03227	B^∗^2705	B^∗^1400, B^∗^1517, B^∗^2701, B^∗^2702, B^∗^2703, B^∗^2704, B^∗^2705, B^∗^2706, B^∗^2709	7.56
VSFIEFVGW	EBNA3	P12977	B^∗^58	B^∗^5701, B^∗^5702	5.08

^1^Antigen accession number from the UniProtKB database. ^2^Experimental restriction profile obtained from epitope databases. ^3^Average population protection coverage (PPC) of PPCs computed for 5 ethnic groups in the USA population (Black, Caucasian, Hispanic, North American natives, and Asians) using the relevant HLA I genetic frequencies [40]. The combination that reached the largest PPC (97.1%) included the CD8 T cell epitopes YVLDHLIVV, YRSGIIAVV, SVRDRLARL, RVRAYTYSK, LPCVLWPVL, and RRIYDLIEL.

**Table 2:** Conserved EBV-specific CD4 T cell epitopes.

Epitope	Antigen gene	AN^1^	HLA II restriction^2^	Predicted HLA II profile	PPC^3^
MLGQDDFIKFKSPLV	BFRF1	P03185	DRB1^∗^0701	DRB1^∗^0901, DRB1^∗^1501, DRB1^∗^0701, DRB1^∗^0405, DRB1^∗^0101, DRB1^∗^0301, DRB5^∗^0101, DRB1^∗^0401	69.85
*AGLTLSLLVI*CSYLFISRG	BHRF1	P03182	DR2	DRB1^∗^1501, DRB5^∗^0101, DRB1^∗^1101, DRB1^∗^0405, DRB1^∗^0401, DRB1^∗^0301, DRB1^∗^1201, DRB1^∗^0802	57.97
LEKQLFYYIGTMLPNTRPHS	BXLF2	P03231	DR51	DRB5^∗^0101, DRB1^∗^1101, DRB1^∗^0401, DRB1^∗^0405, DRB1^∗^1201, DRB1^∗^1501, DRB1^∗^0301, DRB1^∗^0802	57.97
SRRFSWTLFL*AGLTLSLLVI*	BHRF1	P03182	DR2	DRB1^∗^0401, DRB1^∗^0101, DRB1^∗^0901, DRB1^∗^0301, DRB1^∗^0701, DRB1^∗^1201	55.25
SRDELLHTRAASLLY	BARF1	P0CAP6	DRB1^∗^0701	DRB1^∗^0701, DRB1^∗^0101, DRB1^∗^1201, DRB3^∗^0202, DRB1^∗^0901, DRB1^∗^1302 DRB5^∗^0101	42.9
PPVVRMFMRERQLPQ	EBNA6	P03204	HLA class II	DRB1^∗^1101, DRB5^∗^0101, DRB1^∗^0301, DRB1^∗^0401, DRB4^∗^0101	36.88
QQRPVMFVSRVPAKK	EBNA6	P03204	HLA class II	DRB5^∗^0101, DRB1^∗^0802, DRB1^∗^1101, DRB1^∗^0301	29.35
PAQPPPGVINDQQLHHLPSG	EBNA2	P12978	DRB1^∗^0301	DRB1^∗^0301, DRB4 ^∗^0101	17.84
VKLTMEYDDKVSKSH	BMRF1	P03191	DRB1^∗^0301	DRB1^∗^0301	17.84
QKRAAPPTVSPSDTG	EBNA6	P03204	HLA class II	—	0

^1^Antigen accession number from the UniProtKB database. ^2^Experimental HLA II restriction profile obtained from epitope databases. ^3^Population protection coverage (PPC) was computed for the world population using the IEDB Analysis Resources tool with the HLA-DR allele reference set provided by the tool [37]. The italicized sequence is shared by the two epitopes that contain it.

**Table 3:** Experimentally defined conserved EBV-specific B cell epitopes.

Epitope	Antigen (gene)	AN^1^	Epitope location	PDB hit^2^	Flexibility^3^	Access.^4^
SK*APESTTTSPTLNTTGFA*	gp350 (BLLF1)	P03200	Ectodomain	2H6O [422–440]	2.486 (2.672)	59.2 (63.4)
YVFYS*GNGPKASGGD*YCIQS	gp350 (BLLF1)	P03200	Ectodomain	2H6O [282–301]	1.102 (2.004)	31.7 (51.2)
QNPVYLIP*ETVPYIKWDN*	gp350 (BLLF1)	P03200	Ectodomain	2H6O [147–164]	0.618 (1.191)	62.4 (77.5)
SVKTEMLGNEID	gp350 (BLLF1)	P03200	Ectodomain	2H6O [197–208]	−0.347	19.8
QVSLESVDVYFQDVFGTMWC	gp350 (BLLF1)	P03200	Ectodomain	2H6O [122–141]	−0.575	17.5
TNTTDITYVGD	gp350 (BLLF1)	P03200	Ectodomain	2H6O [317–327]	0.121	60.1
PSTSSKLRPRWTFTSPPVTT	gp350 (BLLF1)	P03200	Ectodomain	No	N/A	N/A
QKRAAQRAAGPSVAS	gpB (BALF4)	P03188	Inner domain	No	N/A	N/A
VSGFISFFKNPFGGM	gpB (BALF4)	P03188	Transmembrane	No	N/A	N/A

^1^Accession number from UniProtKB database. ^2^Epitope hit with corresponding PDBs (in bracket sequence hit). Values of ^3^flexibility (arbitrary units) and ^4^solvent accessibility (%) were calculated as explained in Materials and Methods. N/A: not applicable; gp: glycoprotein. We show the italicized regions in B cell epitopes consisting of 9 or more consecutive residues with flexibility ≥ 1 and we show in brackets the corresponding flexibility and accessibility values of these regions.

**Table 4:** Predicted conserved B cell epitopes from EBV envelope proteins.

Epitope	Antigen (gene)	Accession number^1^	PDB^2^	Flex.^3^	Acc. (%)^4^	BLAST hit HMP (%)^5^	BLAST hit human (%)^6^
KLPHWTPTLH	gp42 (BZLF2)	P03205	3FD4:A [45–54]	2.256	80.0	EJZ65106.1 (70.00)	AAH22472.1 (60.00)
NTTVGIELPDA	gpB (BALF4)	P03188	3FVC [307–317]	1.890	67.0	EHM53795.1 (72.73)	XP_011519547.1 (63.64)
SSHGDLFRFSSDIQCP	gpB (BALF4)	P03188	3FVC [32–47]	1.369	69.8	KGF26221.1 (50.00)	XP_011520599.1 (50.00)
FSVEDLFGAN	gL (BKRF2)	P03212	5T1D:B [95–104]	1.505	53.1	EKB09257.1 (65.00)	XP_005271219.1 (70.00)

^1^Accession number from the UniProtKB database. ^2^Epitope location in their corresponding PDBs is shown in brackets. The specific chain is indicated along with the PDB code. ^3^Values of flexibility (arbitrary units) and ^4^solvent accessibility (%) were calculated as explained in Materials and Methods. ^5,6^Accession number of closest epitope BLAST hit in human microbiome proteins and human proteins, respectively (percentage of identity in parenthesis).

**Table 5:** Proposed epitope vaccine ensemble for EBV.

CD8 T cell epitope vaccine component					
Sequence	Antigen	AN^1^	BLAST hit HMP^2^	BLAST hit humans^3^	PPC%^4^

RPPIFIRRL	EBNA3	P12977	EFI49553.1 (55.56)	NP_001182344.1 (66.67)	
SVRDRLARL	EBNA3	P12977	No hit (—)	3HR0 (55.56)	
YVLDHLIVV	BRLF1	P03209	EPH07203.1 (88.89)	XP_011535331.1 (66.67)	
QPRAPIRPI	EBNA6	P03204	EEZ70880.1 (66.67)	AFC01212.1 (55.56)	
LPCVLWPVL	BZLF1	P03206	ETN46892.1 (77.78)	XP_011511695.1 (55.56)	
RVRAYTYSK	BRLF1	P03209	EEY91922.1 (66.67)	CAE46202.1 (55.56)	>95
AYSSWMYSY	EBNA3	P12977	EKB85112.1 (77.78)	EAW88404.1 (66.67)	
VLKDAIKDL	EBNA1	P03211	KXB56071.1 (88.89)	EAX00446.1 (66.67)	
QAKWRLQTL	EBNA3	P12977	EHR35488.1 (66.67)	XP_005255827.1 (77.78)	
RRIYDLIEL	EBNA6	P03204	EDS12420.1 (77.78)	EAW88480.1 (66.67)	
RLRAEAQVK	EBNA3	P12977	No hit (—)	XP_011507142.1 (77.78)	
CYDHAQTHL	BMRF1	P03191	EFF75621.1 (77.78)	CAH10644.1 (66.67)	
SENDRLRLL	BZLF1	P03206	EGG37664.1 (77.78)	EAW88969.1 (77.78)	
YRSGIIAVV	BMRF1	P03191	OFQ99895.1 (88.89)	BAC03504.1 (66.67)	

CD4T cell epitope vaccine component					
Sequence	Antigen	AN^1^	BLAST hit HMP^2^	BLAST hit humans^3^	PPC%^4^

MLGQDDFIKFKSPLV	BFRF1	P03185	EIY33207.1 (46.67)	NP_001284364.1 (53.33)	
AGLTLSLLVICSYLFISRG	BHRF1	P03182	EKN19533.1 (47.37)	EAW92092.1 (52.63)	>81.8
SRDELLHTRAASLLY	BARF1	P0CAP6	EPB87510.1 (66.67)	XP_011514101.1 (66.67)	
PPVVRMFMRERQLPQ	EBNA6	P03204	EFV04068.1 (46.67)	AAP34452.1 (60.00)	

B cell epitope vaccine component					
Sequence	Antigen	AN^1^	BLAST hit HMP^2^	BLAST hit humans^3^	Src.^5^

APESTTTSPTLNTTGFA	gp350 (BLLF1)	P03200	EGY79509.1 (58.82)	NP_001276932.1 (52.94)	E
GNGPKASGGD	gp350 (BLLF1)	P03200	EHM51909.1 (70.00)	NP_055501.2 (70.00)	E
ETVPYIKWDN	gp350 (BLLF1)	P03200	EET62946.1 (50.00)	NP_001193968.1 (50.00)	E
KLPHWTPTLH	gp42 (BZLF2)	P03205	EJZ65106.1 (70.00)	AAH22472.1 (60.00)	P
NTTVGIELPDA	gpB (BALF4)	P03188	EHM53795.1 (72.73)	XP_011519547.1 (63.64)	P
SSHGDLFRFSSDIQCP	gpB (BALF4)	P03188	KGF26221.1 (50.00)	XP_011520599.1 (50.00)	P
FSVEDLFGAN	gL (BKRF2)	P03212	EKB09257.1 (80.00)	XP_005271219.1 (70.00)	P

^1^Accession number from UniProtKB database. ^2,3^Accession number of the closest epitope BLAST hit to human microbiome proteins and human proteins, respectively (percentage of identity in parenthesis). ^4^Population protection coverage (PPC) of the CD8 and CD4 T cell epitope ensemble. ^5^Src., source, whether the epitope derived from an experimental B cell epitope (E) or it was predicted (P).

## Abbreviations

MHC: Major histocompatibility complex
HLA: Human leukocyte antigens
gp: Glycoprotein
nAb: Neutralizing antibody.

## References

[1] Lin Z., Wang X., Strong M. J. (2013). Whole-genome sequencing of the Akata and Mutu Epstein-Barr virus strains. *Journal of Virology*.

[2] Sathiyamoorthy K., Jiang J., Hu Y. X. (2014). Assembly and architecture of the EBV B cell entry triggering complex. *PLoS Pathogens*.

[3] Neves M., Marinho-Dias J., Ribeiro J., Sousa H. (2017). Epstein-Barr virus strains and variations: geographic or disease-specific variants?. *Journal of Medical Virology*.

[4] Young L. S., Rickinson A. B. (2004). Epstein-Barr virus: 40 years on. *Nature Reviews Cancer*.

[5] Vetsika E. K., Callan M. (2004). Infectious mononucleosis and Epstein-Barr virus. *Expert Reviews in Molecular Medicine*.

[6] Faulkner G. C., Burrows S. R., Khanna R., Moss D. J., Bird A. G., Crawford D. H. (1999). X-linked agammaglobulinemia patients are not infected with Epstein-Barr virus: implications for the biology of the virus. *Journal of Virology*.

[7] Shannon-Lowe C. D., Neuhierl B., Baldwin G., Rickinson A. B., Delecluse H. J. (2006). Resting B cells as a transfer vehicle for Epstein-Barr virus infection of epithelial cells. *Proceedings of the National Academy of Sciences of the United States of America*.

[8] Kempkes B., Robertson E. S. (2015). Epstein-Barr virus latency: current and future perspectives. *Current Opinion in Virology*.

[9] Mullen M. M., Haan K. M., Longnecker R., Jardetzky T. S. (2002). Structure of the Epstein-Barr virus gp42 protein bound to the MHC class II receptor HLA-DR1. *Molecular Cell*.

[10] Taylor G. S., Long H. M., Brooks J. M., Rickinson A. B., Hislop A. D. (2015). The immunology of Epstein-Barr virus-induced disease. *Annual Review of Immunology*.

[11] Thorley-Lawson D. A. (2001). Epstein-Barr virus: exploiting the immune system. *Nature Reviews Immunology*.

[12] Strowig T., Brilot F., Arrey F. (2008). Tonsilar NK cells restrict B cell transformation by the Epstein-Barr virus via IFN-gamma. *PLoS Pathogens*.

[13] Hislop A. D., Taylor G. S., Sauce D., Rickinson A. B. (2007). Cellular responses to viral infection in humans: lessons from Epstein-Barr virus. *Annual Review of Immunology*.

[14] Brooks J. M., Long H. M., Tierney R. J. (2016). Early T cell recognition of B cells following Epstein-Barr virus infection: identifying potential targets for prophylactic vaccination. *PLoS Pathogens*.

[15] Amyes E., Hatton C., Montamat-Sicotte D. (2003). Characterization of the CD4^+^ T cell response to Epstein-Barr virus during primary and persistent infection. *The Journal of Experimental Medicine*.

[16] Bu W., Hayes G. M., Liu H. (2016). Kinetics of Epstein-Barr virus (EBV) neutralizing and virus-specific antibodies after primary infection with EBV. *Clinical and Vaccine Immunology*.

[17] De Paschale M., Clerici P. (2012). Serological diagnosis of Epstein-Barr virus infection: problems and solutions. *World Journal of Virology*.

[18] Thompson M. P., Kurzrock R. (2004). Epstein-Barr virus and cancer. *Clinical Cancer Research*.

[19] Ascherio A., Munger K. L. (2015). EBV and autoimmunity. *Current Topics in Microbiology and Immunology*.

[20] Cohen J. I. (2015). Epstein-Barr virus vaccines. *Clinical & Translational Immunology*.

[21] Reche P. A., Keskin D. B., Hussey R. E., Ancuta P., Gabuzda D., Reinherz E. L. (2006). Elicitation from virus-naive individuals of cytotoxic T lymphocytes directed against conserved HIV-1 epitopes. *Medical Immunology*.

[22] Sheikh Q. M., Gatherer D., Reche P. A., Flower D. R. (2016). Towards the knowledge-based design of universal influenza epitope ensemble vaccines. *Bioinformatics*.

[23] Molero-Abraham M., Glutting J. P., Flower D. R., Lafuente E. M., Reche P. A. (2015). EPIPOX: immunoinformatic characterization of the shared T-cell epitome between variola virus and related pathogenic Orthopoxviruses. *Journal of Immunology Research*.

[24] Diez-Rivero C. M., Reche P. A. (2012). CD8 T cell epitope distribution in viruses reveals patterns of protein biosynthesis. *PLoS One*.

[25] Reche P. A., Zhang H., Glutting J. P., Reinherz E. L. (2005). EPIMHC: a curated database of MHC-binding peptides for customized computational vaccinology. *Bioinformatics*.

[26] Zhang Q., Wang P., Kim Y. (2008). Immune epitope database analysis resource (IEDB-AR). *Nucleic Acids Research*.

[27] Li W., Godzik A. (2006). Cd-hit: a fast program for clustering and comparing large sets of protein or nucleotide sequences. *Bioinformatics*.

[28] Federhen S. (2015). Type material in the NCBI taxonomy database. *Nucleic Acids Research*.

[29] Edgar R. C. (2004). MUSCLE: multiple sequence alignment with high accuracy and high throughput. *Nucleic Acids Research*.

[30] Shannon C. E. (1948). The mathematical theory of communication. *The Bell System Technical Journal*.

[31] Garcia-Boronat M., Diez-Rivero C. M., Reinherz E. L., Reche P. A. (2008). PVS: a web server for protein sequence variability analysis tuned to facilitate conserved epitope discovery. *Nucleic Acids Research*.

[32] Reche P. A., Reinherz E. L. (2003). Sequence variability analysis of human class I and class II MHC molecules: functional and structural correlates of amino acid polymorphisms. *Journal of Molecular Biology*.

[33] Stewart J. J., Lee C. Y., Ibrahim S. (1997). A Shannon entropy analysis of immunoglobulin and T cell receptor. *Molecular Immunology*.

[34] Reche P. A., Glutting J.-P., Reinherz E. L. (2004). Enhancement to the RANKPEP resource for the prediction of peptide binding to MHC molecules using profiles. *Immunogenetics*.

[35] Reche P. A., Glutting J. P., Reinherz E. L. (2002). Prediction of MHC class I binding peptides using profile motifs. *Human Immunology*.

[36] Reche P. A., Reinherz E. L. (2007). Prediction of peptide-MHC binding using profiles. *Methods in Molecular Biology*.

[37] Greenbaum J., Sidney J., Chung J., Brander C., Peters B., Sette A. (2011). Functional classification of class II human leukocyte antigen (HLA) molecules reveals seven different supertypes and a surprising degree of repertoire sharing across supertypes. *Immunogenetics*.

[38] Wang P., Sidney J., Kim Y. (2010). Peptide binding predictions for HLA DR, DP and DQ molecules. *BMC Bioinformatics*.

[39] Molero-Abraham M., Lafuente E. M., Flower D. R., Reche P. A. (2013). Selection of conserved epitopes from hepatitis C virus for pan-populational stimulation of T-cell responses. *Clinical & Developmental Immunology*.

[40] Cao K., Hollenbach J., Shi X., Shi W., Chopek M., Fernandez-Vina M. A. (2001). Analysis of the frequencies of HLA-A, B, and C alleles and haplotypes in the five major ethnic groups of the United States reveals high levels of diversity in these loci and contrasting distribution patterns in these populations. *Human Immunology*.

[41] Bui H. H., Sidney J., Dinh K., Southwood S., Newman M. J., Sette A. (2006). Predicting population coverage of T-cell epitope-based diagnostics and vaccines. *BMC Bioinformatics*.

[42] Hubbard S. J., Thornton J. M. (1993). *NACCESS, Computer Program*.

[43] Magrane M. (2011). UniProt Knowledgebase: a hub of integrated protein data. *Database: The Journal of Biological Databases and Curation*.

[44] Altschul S. F., Madden T. L., Schaffer A. A. (1997). Gapped BLAST and PSI-BLAST: a new generation of protein database search programs. *Nucleic Acids Research*.

[45] Peterson J., Garges S., Giovanni M. (2009). The NIH Human Microbiome Project. *Genome Research*.

[46] Glimcher L. H., Kara C. J. (1992). Sequences and factors: a guide to MHC class-II transcription. *Annual Review of Immunology*.

[47] Stern L. J., Calvo-Calle J. M. (2009). HLA-DR: molecular insights and vaccine design. *Current Pharmaceutical Design*.

[48] Szakonyi G., Klein M. G., Hannan J. P. (2006). Structure of the Epstein-Barr virus major envelope glycoprotein. *Nature Structural & Molecular Biology*.

[49] Backovic M., Longnecker R., Jardetzky T. S. (2009). Structure of a trimeric variant of the Epstein-Barr virus glycoprotein B. *Proceedings of the National Academy of Sciences of the United States of America*.

[50] Westhof E., Altschuh D., Moras D. (1984). Correlation between segmental mobility and the location of antigenic determinants in proteins. *Nature*.

[51] Kirschner A. N., Sorem J., Longnecker R., Jardetzky T. S. (2009). Structure of Epstein-Barr virus glycoprotein 42 suggests a mechanism for triggering receptor-activated virus entry. *Structure*.

[52] Sathiyamoorthy K., Hu Y. X., Mohl B. S., Chen J., Longnecker R., Jardetzky T. S. (2016). Structural basis for Epstein-Barr virus host cell tropism mediated by gp42 and gHgL entry glycoproteins. *Nature Communications*.

[53] Smith C., Khanna R. (2015). The development of prophylactic and therapeutic EBV vaccines. *Current Topics in Microbiology and Immunology*.

[54] Balfour H. H. (2014). Progress, prospects, and problems in Epstein-Barr virus vaccine development. *Current Opinion in Virology*.

[55] Sokal E. M., Hoppenbrouwers K., Vandermeulen C. (2007). Recombinant gp350 vaccine for infectious mononucleosis: a phase 2, randomized, double-blind, placebo-controlled trial to evaluate the safety, immunogenicity, and efficacy of an Epstein-Barr virus vaccine in healthy young adults. *The Journal of Infectious Diseases*.

[56] Lin R., Heeke D., Liu H. (2017). Development of a robust, higher throughput green fluorescent protein (GFP)-based Epstein-Barr virus (EBV) micro-neutralization assay. *Journal of Virological Methods*.

[57] Elliott S. L., Suhrbier A., Miles J. J. (2008). Phase I trial of a CD8^+^ T-cell peptide epitope-based vaccine for infectious mononucleosis. *Journal of Virology*.

[58] Hutt-Fletcher L. M. (2015). EBV glycoproteins: where are we now?. *Future Virology*.

[59] Dasari V., Bhatt K. H., Smith C., Khanna R. (2017). Designing an effective vaccine to prevent Epstein-Barr virus-associated diseases: challenges and opportunities. *Expert Review of Vaccines*.

[60] Ponomarenko J., Van Regenmortel M. (2009). B-cell epitope prediction. *Structural Bioinformatics*.

[61] Van Regenmortel M. H. (2009). What is a B-cell epitope?. *Methods in Molecular Biology*.

[62] Reche P. A., Reinherz E. L. (2007). Definition of MHC supertypes through clustering of MHC peptide-binding repertoires. *Methods in Molecular Biology*.

[63] Sun P., Ju H., Liu Z. (2013). Bioinformatics resources and tools for conformational B-cell epitope prediction. *Computational and Mathematical Methods in Medicine*.

[64] Potocnakova L., Bhide M., Pulzova L. B. (2016). An introduction to B-cell epitope mapping and in silico epitope prediction. *Journal of Immunology Research*.

[65] Blythe M. J., Flower D. R. (2005). Benchmarking B cell epitope prediction: underperformance of existing methods. *Protein Science*.

[66] Gao J., Kurgan L. (2014). Computational prediction of B cell epitopes from antigen sequences. *Methods in Molecular Biology*.

[67] Young K. A., Herbert A. P., Barlow P. N., Holers V. M., Hannan J. P. (2008). Molecular basis of the interaction between complement receptor type 2 (CR2/CD21) and Epstein-Barr virus glycoprotein gp350. *Journal of Virology*.

[68] Tanner J. E., Coincon M., Leblond V. (2015). Peptides designed to spatially depict the Epstein-Barr virus major virion glycoprotein gp350 neutralization epitope elicit antibodies that block virus-neutralizing antibody 72A1 interaction with the native gp350 molecule. *Journal of Virology*.

[69] Litwin V., Gumperz J., Parham P., Phillips J. H., Lanier L. L. (1993). Specificity of HLA class I antigen recognition by human NK clones: evidence for clonal heterogeneity, protection by self and non-self alleles, and influence of the target cell type. *The Journal of Experimental Medicine*.

[70] Arnon R., Ben-Yedidia T. (2003). Old and new vaccine approaches. *International Immunopharmacology*.

[71] Whitton J. L., Sheng N., Oldstone M. B., McKee T. A. (1993). A “string-of-beads” vaccine, comprising linked minigenes, confers protection from lethal-dose virus challenge. *Journal of Virology*.

[72] Gupta S., Kapoor P., Chaudhary K., Gautam A., Kumar R., Raghava G. P. (2013). In silico approach for predicting toxicity of peptides and proteins. *PLoS One*.

[73] Varypataki E. M., Benne N., Bouwstra J., Jiskoot W., Ossendorp F. (2017). Efficient eradication of established tumors in mice with cationic liposome-based synthetic long-peptide vaccines. *Cancer Immunology Research*.

[74] Kuai R., Ochyl L. J., Bahjat K. S., Schwendeman A., Moon J. J. (2017). Designer vaccine nanodiscs for personalized cancer immunotherapy. *Nature Materials*.

[75] Brazzoli M., Magini D., Bonci A. (2015). Induction of broad-based immunity and protective efficacy by self-amplifying mRNA vaccines encoding influenza virus hemagglutinin. *Journal of Virology*.

[76] Schwendener R. A. (2014). Liposomes as vaccine delivery systems: a review of the recent advances. *Therapeutic Advances in Vaccines*.

[77] Felnerova D., Viret J. F., Gluck R., Moser C. (2004). Liposomes and virosomes as delivery systems for antigens, nucleic acids and drugs. *Current Opinion in Biotechnology*.

[78] Perrie Y., Kirby D., Bramwell V. W., Mohammed A. R. (2007). Recent developments in particulate-based vaccines. *Recent Patents on Drug Delivery & Formulation*.

[79] Gujer C., Chatterjee B., Landtwing V., Raykova A., McHugh D., Munz C. (2015). Animal models of Epstein Barr virus infection. *Current Opinion in Virology*.

[80] Calcagno C., Puzone R., Pearson Y. E. (2011). Computer simulations of heterologous immunity: highlights of an interdisciplinary cooperation. *Autoimmunity*.

[81] (1997). Proceedings of the IARC working group on the evaluation of carcinogenic risks to humans. Epstein-Barr virus and Kaposi’s sarcoma herpesvirus/human herpesvirus 8. Lyon, France, 17-24 June 1997. *IARC Monographs on the Evaluation of Carcinogenic Risks to Humans*.

[82] Lee A. W., Foo W., Mang O. (2003). Changing epidemiology of nasopharyngeal carcinoma in Hong Kong over a 20-year period (1980-99): an encouraging reduction in both incidence and mortality. *International Journal of Cancer*.

[83] Zhong W., Reche P. A., Lai C. C., Reinhold B., Reinherz E. L. (2003). Genome-wide characterization of a viral cytotoxic T lymphocyte epitope repertoire. *The Journal of Biological Chemistry*.

[84] Lafuente E. M., Reche P. A. (2009). Prediction of MHC-peptide binding: a systematic and comprehensive overview. *Current Pharmaceutical Design*.

