# Supplementary figures and images for: Computer-Aided Design of an Epitope-Based Vaccine against Epstein-Barr Virus
Source: J Immunol Res. 2017 Sep 28;2017:9363750. doi: 10.1155/2017/9363750 (PMC5651165; doi:10.1155/2017/9363750)

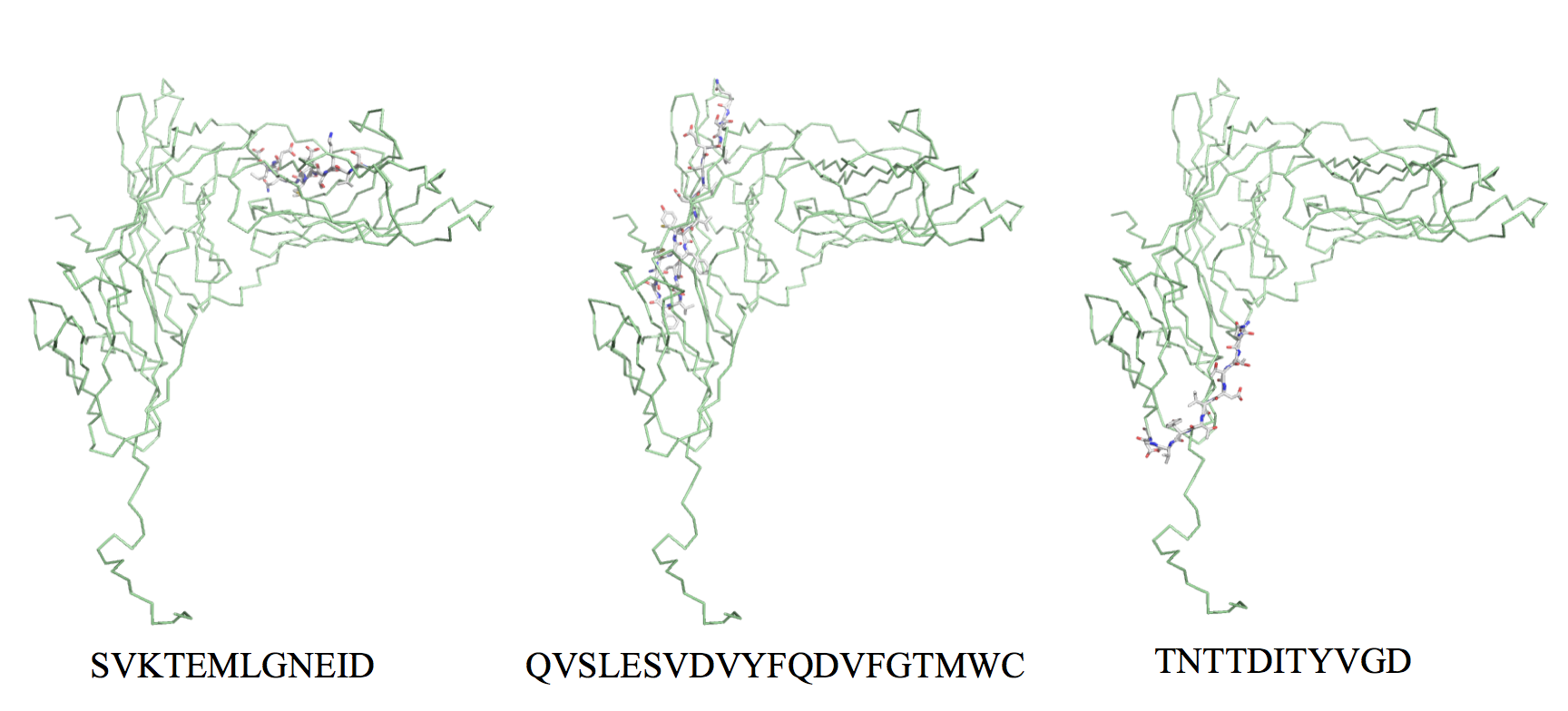

Supplement: Supplementary file 4 [file 9363750.f4.png]
